# Supplementary material for: The interactome of intact mitochondria by cross-linking mass spectrometry provides evidence for coexisting respiratory supercomplexes
Source: Mol Cell Proteomics. 2017 Dec 8;17(2):216–32. doi: 10.1074/mcp.RA117.000470 (PMC5795388; doi:10.1074/mcp.RA117.000470)
Supplement: Supplemental Data [file supp_RA117.000470_133922_0_supp_23978_fzffwf.zip › spectra_annotation/mito_DR_spectra_annotation/336-1-10-1-15-1.pdf]

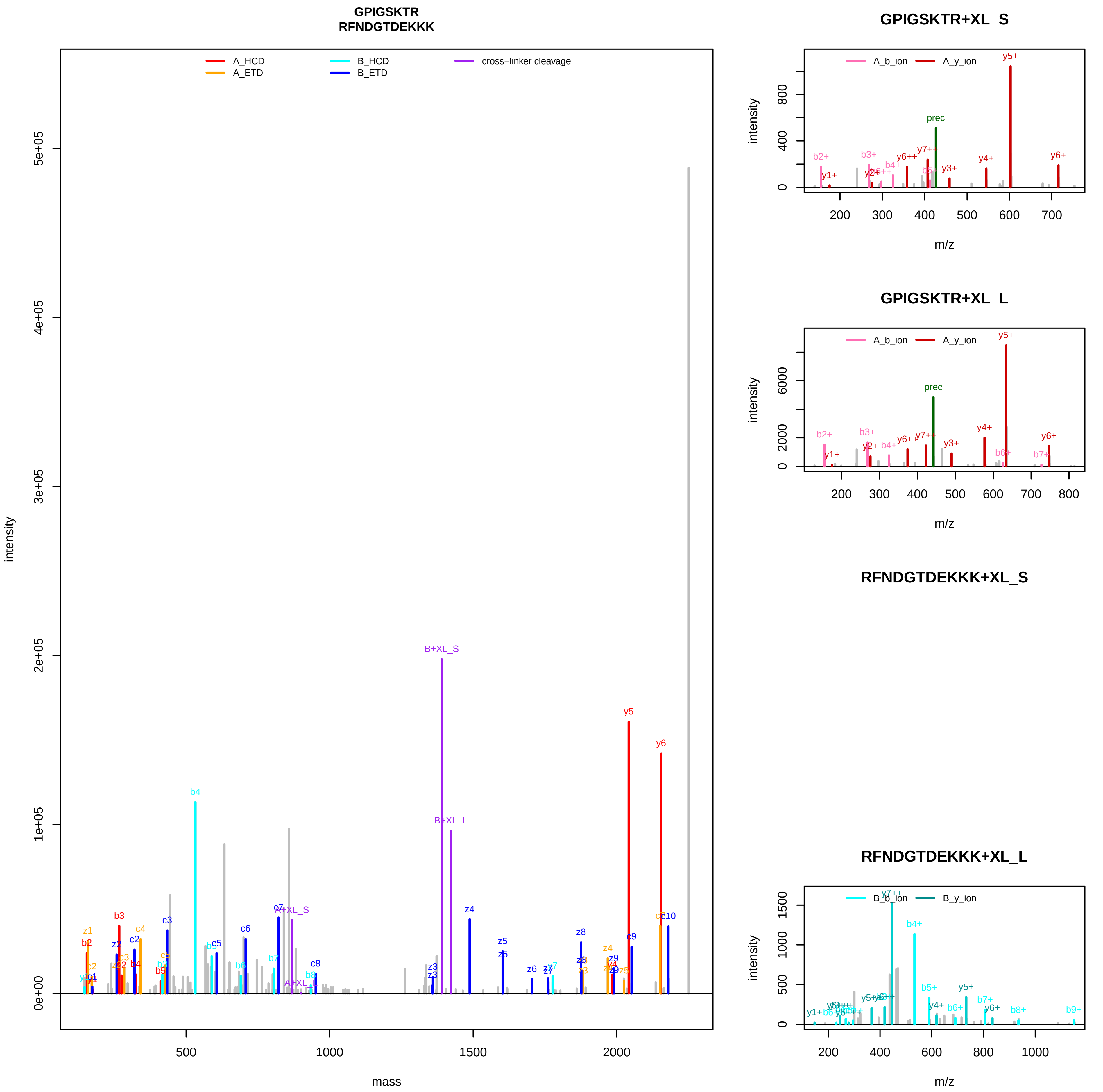
GPIGSKTR
RFNDGTDEKKK
A_HCD
A_ETD
B_HCD
B_ETD
cross−linker cleavage
intensity
mass
0e+00
1e+05
2e+05
3e+05
4e+05
5e+05
500
1000
1500
2000
B+XL_S
B+XL_L
A+XL_S
y5
y6
b4
b3
b2
z1
c3
c6
c7
c8
c9
c10
z4
z5
z6
z7
z8
z9
GPIGSKTR+XL_S
A_b_ion
A_y_ion
prec
y5+
y6+
y4+
y3+
y7++
y6++
b2+
b3+
b4+
y1+
m/z
GPIGSKTR+XL_L
A_b_ion
A_y_ion
prec
y5+
y4+
y6+
y3+
y7++
y6++
b2+
b3+
b4+
b6+
b7+
y1+
y2+
m/z
RFNDGTDEKKK+XL_S
RFNDGTDEKKK+XL_L
B_b_ion
B_y_ion
y7++
b4+
b5+
y5+
b7+
y6+
b8+
b9+
y4+
b6+
y1+
m/z
intensity
